# False Positive PCR for Herpes Simplex Virus (HSV) in Childhood CNS Infections

**Published:** 2012-04-01

**Authors:** M S Sasan

**Affiliations:** 1 Department of Pediatrics, Subspecialty in Pediatric Infectious Diseases, Mashhad University of Medical Sciences, Imam Reza Hospital, Mashhad, Iran

**Keywords:** Herpes simplex virus, HSV, encephalitis, False positive, PCR

In 2010; 12(5):568-571 issue of IRCMJ journal, an article about herpetic encephalitis (HE) in children was published with the following title: Clinical Manifestations, Laboratory Findings and Outcomes of Children with Herpetic Encephalitis in Amirkola Children Hospital, Northern Iran.

The authors found that during 2 years 34% (17 cases) of the 50 patients with clinical diagnosis of encephalitis had positive PCR for HSV, only 28% of their HE cases had altered consciousness but they did not say anything about focal neurologic signs and they did not have any disability or mortality and all of the patients were discharged in good general condition.

I think that there has been a technical problem with HSV PCR in this research because the incidence of HE was unacceptably high and the prognosis was unbelievably good. Their cases must have been cases of viral meningitis with false positive HSV PCR probably due to contamination.

The annual incidence of HE in the whole population was between one in 460000 to 2.2 per million population per year and one-third of all cases occurred in children and adolescents.[1][2][3] Amirkola Children Hospital needs to cover between 4250000 to 18700000 children each year to have such a high number of HE, while we know that the whole population of the Province of Mazandaran (which Babol is the center of it) is around 3000000 and 21.3% of the population are younger than 14 years. Over 90 percent of HSV encephalitis cases have one of the altered mentation and level of consciousness, focal cranial nerve deficits, hemiparesis, dysphasia, aphasia, ataxia, or focal seizures.[1] Even with appropriate diagnosis and treatment, HSV encephalitis has a high rate of severe disability (20%) and mortality (20-30%).[1][3]

Our experience in Pediatric Infectious Diseases Services of Mashhad and Shiraz which are 2 of the first five big cities of Iran and much bigger than Babol showed that in the past 10 years, the mean number of HSV encephalitis cases has been less than 2 patients per year. We have had the problem of the false positive HSV PCR in Nemazee Hospital (Shiraz) at 2000-2002 period, when we encountered with an outbreak of positive HSV PCR among children with aseptic meningitis that we already had discharged them (at very good condition without giving acyclovir) with clinical diagnosis of viral meningitis, many of them were mumps meningitis.
